# Applications of Machine Learning in the Diagnosis and Prognosis of Patients with Chiari Malformation Type I: A Scoping Review

**DOI:** 10.3390/children12020244

**Published:** 2025-02-18

**Authors:** Solonas Symeou, Marios Lampros, Panagiota Zagorianakou, Spyridon Voulgaris, George A. Alexiou

**Affiliations:** Department of Neurosurgery, University Hospital of Ioannina, 45500 Ioannina, Greece; md07011@uoi.gr (S.S.); m.lampros@uoi.gr (M.L.); panzagorian@gmail.com (P.Z.); svoulgar@uoi.gr (S.V.)

**Keywords:** machine learning, deep learning, Chiari malformation, CMI patients, CMI diagnosis, CMI prognosis

## Abstract

**Background:** The implementation of machine learning (ML) models has significantly impacted neuroimaging. Recent data suggest that these models may improve the accuracy of diagnosing and predicting outcomes in patients with Chiari malformation type I (CMI). **Methods**: A scoping review was conducted according to the guidelines put forth by PRISMA. The literature search was performed in PubMed/MEDLINE, SCOPUS, and ScienceDirect databases. We included observational or experimental studies focusing on the applications of ML in patients with CMI. **Results:** A total of 9 articles were included. All the included articles were retrospective. Five out of the nine studies investigated the applicability of machine learning models for diagnosing CMI, whereas the remaining studies focused on the prognosis of the patients treated for CM. Overall, the accuracy of the machine learning models utilized for the diagnosis ranged from 0.555 to 1.00, whereas the specificity and sensitivity ranged from 0.714 to 1.00 and 0.690 to 1.00, respectively. The accuracy of the prognostic ML models ranged from 0.402 to 0.820, and the AUC ranged from 0.340 to 0.990. The most utilized ML model for the diagnosis of CMI is logistic regression (LR), whereas the support vector machine (SVM) is the most utilized model for postoperative prognosis. **Conclusions:** In the present review, both conventional and novel ML models were utilized to diagnose CMI or predict patient outcomes following surgical treatment. While these models demonstrated significant potential, none were highly validated. Therefore, further research and validation are required before their actual implementation in standard medical practice.

## 1. Introduction

Chiari malformations (CMs) are a group of congenital abnormalities affecting the posterior cranial fossa (PCF), the craniovertebral junction (CVJ), and the anatomical structures within those areas, including the cerebellum, pons, medulla oblongata, and spinal cord [1,2,3]. The hallmark feature of CM is the herniation of the cerebellar tonsils through the foramen magnum (FM), though other pathological features—such as brainstem compression, nerve distortion, CSF-flow dynamic obstruction, and syrinx cavities, may also be present [4,5]. Classically, four types of CMs (I, II, III, IV) have been described in the literature, primarily based on the degree of cerebellar descent. However, advancements in neuroradiology have led to a more refined diagnosis, expanding this number to nine (CM1, 2, 3, 4, 0, 0.5, 1.5, 3.5, 5) [6,7,8]. Despite these developments, standardized classification methods, guidelines and frameworks remain limited or nonexistent.

Among all Chiari malformations, type I (CMI) is the most common, with a prevalence ranging from 0.5 to 3.5% in the general population and approximately 1% in the pediatric population [1,6,9]. CMI is classically defined as the caudal displacement of the cerebellar tonsils greater than 5 mm below McRae’s line (a line extending from the basion to the opisthion) on MRI. However, due to the lack of consensus in radiological assessment and classification, ectopia between 3 and 5 mm when accompanied by syringomyelia or a peg-like appearance of the cerebellar tonsils is also considered diagnostic for CMI [10]. Accompanying clinical signs are not specific, making diagnosis challenging. As a result, neuroradiologists and neurosurgeons may struggle to diagnose and accurately differentiate CMI from other types of Chiari Malformation or neurological disorders with similar clinical presentation [11,12,13,14]. In this context, machine learning models could serve as a valuable tool for physicians managing these patients, aiding in more accurate diagnosis and classification [15,16].

Machine learning (ML), a subset of artificial intelligence (AI), focuses on developing algorithms that enable computers to learn from data and make predictions or decisions based on images and other inputs. Unlike traditional programming, where humans code explicit instructions for specific tasks, ML algorithms identify patterns within complex data and improve their performance over time without being explicitly programmed for every scenario [17,18]. ML models have significant applications in the medical field, particularly neurosurgery, including neuro-oncology, neurotrauma, functional, vascular and pediatric neurosurgery. These applications focus on enhancing diagnostic and prognostic accuracy [15,19]. ML models optimize their performance based on the specific cases they are applied to, often detecting complex patterns in large datasets. Among the many ML models available today, the two most prominent categories are “supervised” and “unsupervised” models [20]. The key difference between these models lies in the type of data they utilize. Supervised models work with predefined or “labeled” data, meaning that the training data are tagged with the correct outcome (e.g., patient A has a definitive CMI diagnosis) [21]. During training, the model’s performance is periodically evaluated on a separate validation dataset to fine-tune its accuracy and prevent overfitting. Overfitting occurs when a model memorizes training data too well, leading to poor performance on new, unseen cases [22]. This limitation is detrimental because it reduces the model’s generalizability and clinical utility in medical practice [22]. Despite this challenge, supervised models can serve as powerful tools for diagnosing and prognosing CMI as well as differentiating Chiari Malformation type I from other Chiari Malformations. On the other hand, unsupervised models analyze unlabeled data (e.g., patients without definitive CMI diagnosis) and aim to identify hidden structures, patterns, or relationships within the input data [23]. These models can uncover novel insights that may not be immediately apparent, aiding in the classification and understanding of CMI.

This study represents the first scoping review that systematically maps the current research on the application of machine learning models in CMI, emphasizing the clinical implications of this emerging technology.

## 2. Methods

A scoping review was conducted according to the Preferred Reporting Items for Scoping Reviews (PRISMA-ScR) guidelines to assess the applicability of machine learning models in diagnosing and prognosticating Chiari malformation type I (CMI).

### 2.1. Literature Search

A comprehensive search was carried out across three databases: PubMed/MEDLINE, SCOPUS, and ScienceDirect. The final literature search was conducted on 10 September 2024.

### 2.2. Search Algorithm

The following search string query was applied to searches in PubMed/MEDLINE and SCOPUS: («machine learning” OR “deep learning” OR “ensembled learning” OR “artificial neuronal network” OR “naive Bayes” OR “decision trees” OR “k-nearest neighbor” OR “artificial intelligence”) AND (“chiari malformation” OR “posterior cranial fossa overcrowding”). A similar but modified algorithm was applied in ScienceDirect.

### 2.3. Eligibility Criteria

We included primary research studies written in English. We did not apply any restrictions regarding patient demographics, such as age, sex, or ethnicity. Eligible studies focused solely on the use of machine learning for diagnosing and prognosing CMI, regardless of the specific model utilized. The term “application” refers to the implementation of ML algorithms for diagnostic and prognostic purposes.

### 2.4. Exclusion Criteria

We excluded review articles, meta-analyses, studies in languages other than English, and animal studies. Additionally, studies with irrelevant outcomes or those employing diagnostic or prognostic algorithms unrelated to artificial intelligence (AI) were not included.

### 2.5. Data Collection and Extraction

Two investigators (S.S. and L.M.) independently and preliminarily screened the titles and abstracts, with a third investigator (Z.P.) resolving disagreements on study eligibility. Full-text articles were then retrieved and screened according to the established eligibility criteria.

Data from each included article were extracted and recorded in a table using Microsoft Excel. The data of interest included the following: year of publication, study design, sample size/number of patients, age, imaging modality used for diagnosing CMI, number of images taken, imaging parameters, and diagnostic ground truth. Furthermore, we recorded the types of machine learning models, their specific purpose, the input/output data and their primary outcomes. Information regarding training and testing sets and validation methods was also retrieved. When available, we also extracted performance metrics such as accuracy, sensitivity, specificity, and Area Under the Curve (AUC) calculated on the testing set of each model.

### 2.6. Statistical Analysis

The included studies were highly heterogeneous in terms of age, sex, population size, types of ML models, input data, and primary outcomes. Therefore, statistical analysis and tests were not performed.

## 3. Results

**Figure 1:**
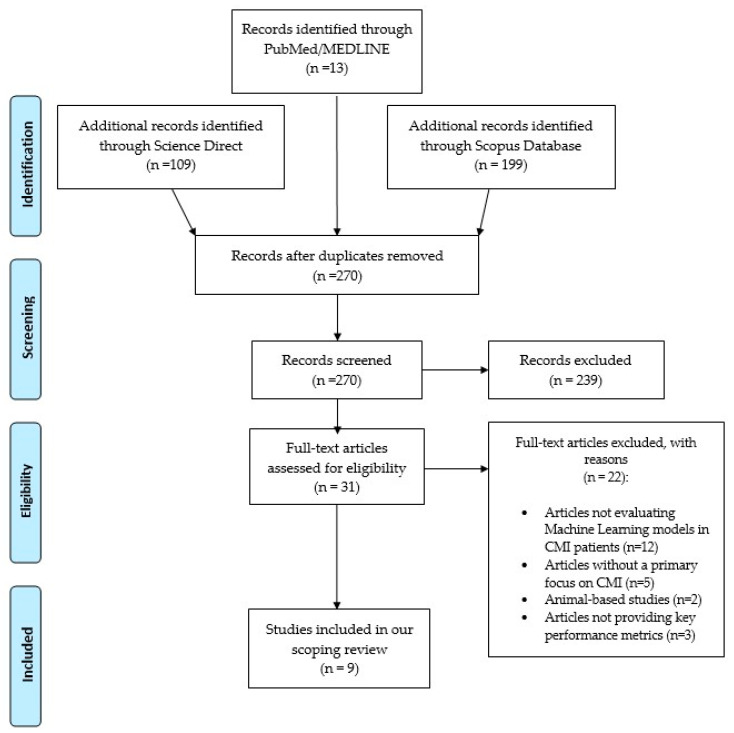
PRISMA flowchart for the selection of eligible studies.

In the preliminary search of the literature, a total of 321 results were obtained from Medline, Scopus, and ScienceDirect databases. After removing 51 duplicates, the remaining 270 articles were screened based on the titles and abstracts. Subsequently, three independent authors (S.S., L.M., Z.P.) assessed the full text of 31 eligible articles. Of these, 12 were excluded because they did not specifically evaluate the application of machine learning in Chiari patients, but instead, they focused on other modalities, such as statistical logistic regression and other advanced MRI techniques. Five studies were excluded because they reviewed the general use of machine learning models in neurosurgery without a primary focus on CMI, and two studies were animal-based. Ultimately, three studies, although related to the topic, did not provide key performance metrics such as sensitivity, specificity, accuracy, and Area Under the Curve. Finally, nine articles [24,25,26,27,28,29,30,31,32] were included in this review. Figure 1 illustrates the study selection for this scoping review.

Of the included articles, five focused on the applicability of machine learning models for diagnosing CM patients (Table 1). The remaining four studies evaluated the ability of ML models to predict postoperative outcomes in the same patient cohort (Table 2). All studies were retrospective.

### 3.1. Input Variables

A qualitative interpretation of the input data used in the ML models was performed. Specifically, the input data included demographic information (age, sex, race), clinical data (symptoms, ASA score, physical component scale of the SF-12 questionnaire, operative time), MR images, and morphometric parameters extracted from the imaging techniques (MRIs). MR images were taken in the sagittal [24,28,30,32] and midsagittal planes [25,26,27], with the preferred sequence being either T1-weighted (T1W) [24,25,26,27,32] or T1-fluid-attenuated inversion recovery (T1-FLAIR) [28,30]. Additionally, CPC-MRI (cine phase contrast) was used in a study to calculate the MRI scores, which were later included in the input data of some models [30]. Two studies did not provide further details regarding the imaging axis or sequence utilized [29,31]. The most commonly used input data were “morphometric parameters”, included in 6 out of 9 studies [24,26,27,30,31,32] (66.7%), followed by MR-Images [25,29,30], and clinical and demographic data, which were used in 3 out of 9 studies [29,30] (33.3%).

Interestingly, 5 out of 9 studies (55.5%) employed a combination of parameters, either combining various morphometric parameters (ranging from two to seven) [26,27] or incorporating a blend of demographic, clinical and morphometric parameters [29,30,32]. Due to the heterogeneity in size and type of input data, a more in-depth analysis was not conducted. A qualitative visualization of the input variables is presented in Supplementary Table S2.

### 3.2. Validity

The authors provided details regarding the internal validation of all 46 ML models. Validation methods included either cross-validation or leave-one-out validation. Cross-validation was more widely used, being the method of choice for 84.8% of the models (39 out of 46 models) [25,26,27,28,29,30,31]. In contrast, leave-one-out validation was used for 15.2% of the models (7 out of 46 models) [24,32]. None of the 46 models underwent external validation (Table 1 and Table 2, Supplementary Table S2).

### 3.3. ML Models Designed to Diagnose CHI

A total of 25 ML models were designed to determine whether patients have Chiari malformation type I. Of these, one was a hybrid model, combining two conventional models, whereas all the others were single-type, conventional algorithms [24]. Specifically, the hybrid model utilized a combination of Naive-Bayes (NB) and Binary Trees Classification (BT), while the other ML models included Convolutional Neural Network (CNN), Extreme Gradient Boosting (XGBoost), Support Vector Machine (SVM), Stochastic Gradient Boosting (SGB), NB, Quadratic Dominant Analysis (QDA), Bagged CART (BC), Linear Discriminant Analysis (LDA), Logistic Regression (LR), Random Forest (RF), Decision Trees (DT) and K-Nearest-Neighbors (KNN). CNN was the most popular, comprising 28% of the diagnostic models (7 out of 25) [25,28], followed by LR [22,23] and XGBoost [26], accounting for 12.0% (3 out of 25) and 8.0% (2 out of 25), respectively (Table 1 and Supplementary Table S2).

### 3.4. Diagnostic Models’ Performance

The accuracy of the ML models for diagnosing CMI ranged from 0.555 [26] to 1.00 [26], whereas the sensitivity and specificity varied from 0.690 [26], 1.00 [26] and 0.714 [28] to 1.00 [25,27,28], respectively. More specifically, the LR model utilizing the distance from the dens axis to the posterior margin of the FM in a study had the lowest accuracy, calculated at 0.555 [26]. In contrast, other models within the same study that used either a single or a combination of morphometric parameters achieved perfect accuracy (1.00). The single-parameter models that performed at this level included SGB, XGBoost, BC, RF, and LR, all of which used tonsillar herniation (TH) as the input parameter. Additionally, models incorporating multiple parameters, such as XGBoost (combining four and seven parameters), Bagged CART (combining three, four, five and seven parameters), and RF (three, four, five, six and seven morphological features), demonstrated similar high performance. Regarding sensitivity, the lowest (0.690) and highest (1.00) values were observed in a single-parameter RF model, which utilized the clivus canal angle and TH, respectively [26]. The lowest specificity (0.714) was reported for the ResNet50 model (dropout rate: 0.50), which analyzed non-cropped MR images [26]. In contrast, the highest specificity (1.00) was achieved by multiple models, including CNN models (one using original-sized cervical MR images and another focusing on the craniocervical junction), a RF model using the TH as a single parameter, and two VGG19 models (nonaugmented models with dropout rates of 0.5 and 0.8) utilizing cropped and uncropped MR images [25,26,28]. The AUC values ranged from 0.890 to 1.00. The ResNet50 model, with a 0.50 dropout rate, exhibited the lowest AUC value, whereas the highest (1.00) was reported for the two CNN models, as well as VGG19, with a dropout rate of 0.80 [25,28]. (Table 1 and Supplementary Table S2)

Table 3 compares the performance of various ML models across different categories in terms of sensitivity and specificity. Deep learning-based models, like CNN and ResNet50, show high sensitivity (up to 0.970) and specificity (up to 1.00), with models such as VGG19 + Augmentation exhibiting excellent performance across both metrics. Tree-based models, such as RF and DT, demonstrate variability in sensitivity (0.690–1.00) and specificity (0.790–1.00), indicating a more diverse performance. Statistical-based models, including Logistic Regression and Linear Discriminant Analysis, also show strong sensitivity and specificity, with LR reaching up to 0.920 and 0.840, respectively. Kernel-based models, like SVM, display high sensitivity (0.880–0.930) and specificity (0.840–0.900), while the hybrid model demonstrates a sensitivity of 0.880 and a specificity of 0.848.

### 3.5. ML Models Designed to Predict Postoperative Outcomes in CMI Patients

A total of 21 models were developed to predict postoperative outcomes in patients who underwent surgery for Chiari Malformation type I. A single model combined CLAM with a ResNet50 algorithm [30], while the remaining were conventional ML models. The most frequently used was the SVM, which accounted for 33.3% (7 out of 210) of all prognostic models [31,32]. Logistic Regression was the second most common, representing 14.3% (3 out of 21) of all prognostic MLs [29,30]. (Table 2 and Supplementary Table S2)

### 3.6. Post-Treatment Outcomes of CMI Patients

In three out of the four studies examining the role of ML models in the postoperative period of CMI patients, the primary outcomes assessed were symptom improvement and recurrence [30,31,32]. In a single study, the focus was on the risk of readmission or reoperation following posterior fossa decompression surgery [29].

### 3.7. Predictive Models’ Performance

The accuracy of the ML models trained to predict postoperative outcomes in CMI patients ranged from 0.402 [30] to 0.840 [31]. Sensitivity varied from 0.450 [30] to 0.725 [30], while specificity ranged from 0.233 [30] to 0.650 [30]. The lowest accuracy was observed in a logistic regression model utilizing age and sex as input data [30], whereas the highest accuracy was achieved by an XGBoost model incorporating 24 morphometric parameters [31]. The model with the lowest sensitivity was a LR model using age and sex as input data [30], while the highest sensitivity was recorded for the hybrid model [30]. When symptoms were used as input data for the LR model, specificity was the lowest (0.233) [30]; however, incorporating cerebellar ectopia combined with the physical component scale of the SF-12 questionnaire improved the specificity to 0.650 [30]. Additionally, the AUC values ranged from 0.340, as seen for a LR model in a study using age and sex, to 0.990 for a Random Forest model predicting the 30-day reoperation risk [29,30]. The latter model utilized both demographic and clinical data as input variables (Table 2 and Supplementary Table S2).

Table 4 presents a comparison of the performance of various ML models throughout different categories based on AUC/AUROC. Tree-based models (RF, XGBoost) show outstanding performance with AUC values ranging from 0.820 to 0.990. Decision Trees also demonstrate solid prognostic abilities with AUC ranging from 0.837 to 0.916. Statistical-based models, including Logistic Regression and K-Nearest Neighbors, exhibit more variability in performance, with LR ranging from 0.340 to 0.820 and KNC achieving high AUC values of 0.921 to 0.940. Kernel-based models, such as SVM, perform with AUC values ranging from 0.870, indicating a significant ability to discriminate different outcomes in patients with CMI. The hybrid model (CLAM + ResNet50) shows the lowest performance with an area under the curve of 0.680, suggesting that it may be less effective for prognostic tasks compared to the other models.

## 4. Discussion

This is the first scoping review to explore the potential applications of machine learning in Chiari malformation type I. Nine articles were included after a thorough screening process, as described in “Methods” [24,25,26,27,28,29,30,31,32]. Two key areas were identified regarding the use of ML in CMI: (1) ML for diagnosis and (2) ML for prognosis in patients undergoing surgery for the condition. Both hybrid and conventional ML models were utilized, with the conventional models being more prevalent [24,30]. The maximum area under the curve (AUC) for diagnosis and prognosis of CMI were 0.800 and 0.990, respectively. These results highlight the promising potential of integrating ML technology into the management of CMI, offering valuable support to physicians in making informed decisions. Moreover, such integration could enhance healthcare efficiency by reducing time and costs, ultimately improving the quality of care to patients [29].

The most used algorithm in the reviewed studies was Support Vector Machine (SVM), which accounted for 17.4% of all the models (8 out of 46), closely followed by Convolutional Neural Networks (CNN), representing 15.2% of the models (7 out of 46). A fundamental feature of ML models is the input data, which varies in size and type depending on the specific problem the models aim to address. In this review, the most frequently utilized variables were morphometric parameters (featuring in 6 out of 9 studies). These parameters, primarily derived from MR images [24,26,27,30,31,32], include tentorium length, supraoccipital length, anteroposterior diameter of the foramen magnum, clivus length, posterior cranial fossa area and osseous PCF area. A more comprehensive list of these parameters can be found in Supplementary Table S2. In addition to morphometrics, clinical, demographic, and MRI-based variables were used as input features in 3 out of 9 studies (Supplementary Table S2).

### 4.1. Machine Learning Models for Diagnosing CMI

Chiari malformation type I is primarily a genetic deformity of the posterior cranial fossa and hindbrain, which leads to the caudal descent of the cerebellar tonsils. It occurs in approximately 1 in 1000 births, with a minor female predominance [9]. Additionally, other etiologies, such as arteriovenous malformations (AVMs), meningiomas, arachnoid cysts, and supratentorial over-drainage, can cause acquired forms of CMI. These acquired forms tend to be more symptomatic, with delayed onset of symptoms, including paroxysmal occipital and cervical headaches, nausea, weakness and sensory deficits, which negatively affect the quality of life [33,34,35]. Therefore, prompt diagnosis and differentiation are critical. While clear and robust clinical guidelines for CMI remain to be established, efforts are underway to incorporate ML models in clinical practice in an attempt to enhance diagnostic accuracy and sensitivity [36]. Notably, several ML models achieved promising results. In a study, single-output models, including XGBoost, SGB, BC, RF, and LR, demonstrated perfect accuracy (1.00) [26]. Similarly, in another study, CNN models achieved accuracy values of 0.970 and 0.960 (Table 1). Furthermore, Urbizu et al. even tested the models on independent cohorts of patients listed as CMI (Table 1) as well as CMI with moderate TH, CM1.5, CM2, and CM0 (Supplementary Table S1) [27]. Surprisingly, accuracy in these cohorts ranged from 0.500 for a Naïve Bayes model (NB) applied to CM0 patients to 1.00 for a QDA in the CMI cohort (Supplementary Table S1 and Table 1). When models across different categories in terms of sensitivity and specificity were compared, deep learning models (CNN, ResNet50 and VGG19) exhibited excellent performance across both metrics, often reaching sensitivity values as high as 0.971 and specificity near 1.00. These results suggest that deep learning models are particularly effective and appear to outperform other categories of ML in tasks requiring optimal accuracy (Table 3).

### 4.2. Machine Learning Prognosing CMI

Owing to the dynamic nature of the CSF, an obstruction to its flow, as observed in CM patients, can lead to the formation of a syrinx [37]. This is a gliosis-lined, fluid-filled sac that most commonly occurs at the level of C2-T9, causing damage to the decussating fibers of the lateral spinothalamic tracts at the anterior white commissure [5]. Consequently, the sensation of pain and temperature—essential for survival and protection—is impaired. As the disease progresses and the lesion expands, it may affect the anterior horns, leading to lower motor neuron signs and diminished quality of life. Early treatment is necessary for impeding the progression of symptoms and disease in general [5]. Two therapeutic approaches are discussed in the literature, with both conservative and surgical strategies being employed. Conservative management is recommended for patients with mild or absent symptoms and involves treatments such as anti-inflammatory drugs, myorelaxants, analgesics, multivitamins, and other non-pharmaceutical interventions, such as swimming, Pilates, osteopathy, and psychological support [3]. In contrast, symptomatic patients often undergo posterior fossa decompression surgery, which aims to alleviate pressure at the foramen magnum and the cervical spine and restore CSF flow [37,38]. To date, there are no clearly defined guidelines for managing these patients, and the majority of treatment decisions are based on physicians’ experience [39]. Moreover, the risk of failed operations or adverse events remains high. Given these challenges, the development of robust algorithms that can accurately inform physicians regarding the outcomes would be highly beneficial. Table 2 presents efficient models for this task, including an RF algorithm that appears to predict the risk of reoperation and hospital readmission using demographic or clinical data, with AUC of 0.990 and 0.921, respectively [29]. A KNC model achieved a similarly high AUC ranging from 0.921 to 0.944 [29] for the same outcomes and input variables. XGBoost and SVM demonstrated promising effectiveness, with AUC at 0.880 and 0.870 for the outcomes “improvement or no improvement” [31]. One study using Logistic Regression demonstrated lower AUC values, ranging from 0.340 to 0.400 when considering age, sex, symptoms, or CINE score as input data [30]. Different categories of ML models exhibit varying capabilities. For instance, tree-based models (DT, RF, XGBoost) appeared to demonstrate the highest reliability for predicting the outcomes of CMI patients, with AUC values ranging from 0.820 to 0.990. Other categories, such as kernel-based models, achieved a solid area under a curve of 0.870, comparable to tree-based models. Meanwhile, statistical-based and hybrid models had lower AUC (0.340 to 0.820 and 0.680, respectively), indicating relatively poorer performance (Table 4). In summary, tree-based models and KNC offer superior AUC, making them highly reliable for prognostic modeling on the basis of Chiari Malformation type-I patients.

### 4.3. Machine Learning Potentials

Machine learning (ML), a branch of artificial intelligence (AI), enables computers to learn from data and make predictions or decisions without explicit programming [40]. This revolutionized the provision of advanced tools for diagnosis, treatment, and beyond [18]. By processing large volumes of medical data—such as patient records, imaging scans and laboratory results- ML algorithms can detect patterns and anomalies that may indicate disease. As a result, treatments have become more precise, leading to improved patient outcomes [41]. With its vast potential, ML is an emerging field that healthcare professionals and researchers should actively explore and integrate into clinical practice [33,34].

### 4.4. Limitations

This scoping review has several limitations. The retrospective design of the included studies, as well as the high heterogeneity in age and size of the population, machine learning algorithms, validation methods, and outcomes, did not provide fertile ground for further quantitative analysis. In some studies, patients were drawn from preexisting databases, whereas in others, complex CMI cases were excluded, consequently predisposing to generalization bias [29,30]. Performance metrics such as Accuracy, Sensitivity, Specificity, and Area Under the Curve need to be improved in some studies [24,26,27,28,29,31,32]. Hence, the evaluation of the effectiveness and comparison of ML models with respect to Chiari Malformation Type-I was accomplished using only the available data provided. Furthermore, the validity of the models is still uncertain. The small samples of patients used in training and internal validation of the ML models, the lack of randomization and, most importantly, the absence of external validation are some of the major limiting factors that need to be resolved. Specifically, the lack of external validation poses a significant obstacle, as models trained on single-institution datasets may not generalize across diverse patient populations, imaging protocols, and clinical settings. This reduces their reliability and limits their adoption in real-world practice, where variability is inevitable. Also, without rigorous validation, there is a heightened risk of biased predictions and misdiagnoses, ultimately leading to ineffective treatments and mistrust among patients toward healthcare providers.

### 4.5. Future Perspectives

It is crucial to emphasize the urgent need for diagnostic and prognostic algorithms for Chiari patients, the implementation of which will significantly impact the lives of both patients and physicians. Machine learning holds significant potential for improvement. Automated radiology screening could enhance MRI interpretation by detecting tonsillar herniation and CSF flow abnormalities with greater accuracy and less intra and interobserver variability, facilitating early and accurate diagnosis. Moreover, predictive models for postoperative risk stratification could assist in identifying patients at higher risk of complications, hospital readmission or reoperation, thereby optimizing surgical decision-making and ensuring that only those most likely to benefit undergo invasive treatment. Despite the undeniable advantages of these models in clinical practice, future research should prioritize multi-institutional collaborations and federated learning approaches that maintain privacy and standardization of data from diverse demographics to augment generalizability. Real-time prospective validation studies are also another strategy that could strengthen the model’s credibility and allow for more seamless external validation.

## 5. Conclusions

This study showcases the promising potential that conventional and hybrid ML has for CMs and suggests that the introduction of such models could be beneficial for healthcare. Despite the promising results of these modalities in terms of accuracy, specificity, and sensitivity, the insufficient number of utilized populations and the lack of external validation still hinder the induction of such methods in clinical practice. Hence, further research that would gradually improve the performance of such models and eliminate these limiting factors to the maximum extent possible is necessary.

## Figures and Tables

**Table 1:** The table demonstrates the included studies focusing on diagnosing patients with Chiari Malformation.

Author	Year	No. of Patients	Age	Imaging Modality	No. of Images	SEQ	D.R	ML Model	Purpose	Output Data	Outcome	Validation	Test Size	Acc	Sen	Spe	AUC
Mesin et al. [24]	2019	50	Pediatric Pts and Adult Control (NA)	MRI	50	T1W (S)	N/A	NB–BT	Diagnosis of CM1 and Decision for Surgery Technique	3	Healthy/Mild Disease/Severe Disease	Leave-Oue-Out	N/A	0.919	0.880	0.848	N/A
Lin et al. [25]	2022	353	45.42 yrs	MRI	675	T1W (MS)	E	CNN Dpr:0.2 CI	Diagnosis of CM1	2	CM1/Normal	5-Fold-Cross-Validation	100	0.960 (0.940, 0.970)	0.920 (0.880, 0.940)	1.00 (1.00, 1.00)	1.00 (1.00, 1.00)
								CNN Dpr:0.2 CVI					100	0.970 (0.950, 0.980)	0.970 (0.910, 1.00)	1.00 (0.960, 1.00)	1.00 (1.00, 1.00)
Tetik et al. [26]	2021	200	29.92 yrs	MRI	200	T1W (MS)	E	XGBoost (S)	Diagnosis of CM1	2	CM1/Control	5-Fold-Cross-Validation	N/A	1.00, 0.845, 0.775, 0.830, 0.780, 0.805, 0.770, 0.785, 0.790, 0.840, 0.810	N/A	N/A	N/A
								SGB (S)						1.00, 0.820, 0.820, 0.815, 0.735, 0.845, 0.800, 0.755, 0.785, 0.850, 0.810			
								BC (S)						1.00, 0.855, 0.830, 0.835, 0.865, 0.875, 0.825, 0.785, 0.835, 0.880, 0.815			
								RF (S)						1.00 (1.00, 1.00), 0.860 (0.812, 0.900), 0.835 (0.784, 0.886), 0.840 (0.789, 0.891), 0.870 (0.823, 0.917), 0.880 (0.835, 0.925), 0.830 (0.778, 0.882), 0.795 (0.739, 0.851), 0.835 (0.784, 0.886), 0.880 (0.835, 0.925), 0.815 (0.761, 0.869)	1.00 (0.964, 1.00), 0.790 (0.697, 0.865), 0.740 (0.643, 0.823), 0.740 (0.643, 0.823), 0.800 (0.708, 0.873), 0.850 (0.765, 0.914), 0.750 (0.653, 0.831), 0.750 (0.653, 0.831), 0.810 (0.719, 0.882), 0.840 (0.753, 0.906), 0.690 (0.590, 0.779)	1.00 (0.964, 1.00), 0.930 (0.861, 0.971), 0.930 (0.861, 0.971), 0.940 (0.874, 0.978), 0.940 (0.874, 0.978), 0.910 (0.836, 0.958), 0.910 (0.836, 0.958), 0.840 (0.753, 0.906), 0.860 (0.776, 0.921), 0.920 (0.848, 0.965), 0.940 (0.874, 0.978)	
								LR (S)						1.00, 0.740, 0.680, 0.690, 0.660, 0.690, 0.700, 0.570, 0.675, 0.555, 0.785	N/A	N/A	
								XGBoost (C)	Diagnosis of CM1	2	CM1/control	5-Fold-Cross-Validation	N/A	0.990, 0.955, 1.00, 0.995, 0.995, 1.00			
								SGB (C)						0.840, 0.840, 0.895, 0.975, 0.890, 0.775			
								BC (C)						0.980, 1.00, 1.00, 1.00, 0.995, 1.00			
								RF (C)						0.980, 1.00, 1.00, 1.00, 1.00, 1.00			
								LR (C)						0.815, 0.82, 0.815, 0.835, 0.865, 0.875			
Urbizu et al. [27]	2018	245	35.6 ± 7.6 yrs	MRI	167	T1W (MS)	NS	LDA	Diagnosis of CM1	2	CM1/control	4-Fold-Cross-Validation	167	N/A	0.910, 0.930, 0.910, 0.910 0.920, 0.920	0.820, 0.840, 0.860, 0.880, 0.880,0.880	N/A
								QDA							0.910, 0.920, 0.920, 0.910, 0.950, 0.930	0.820, 0.840, 0.860, 0.880, 0.860, 0.880	
								NB							0.860, 0.870, 0.900, 0.870, 0.890, 0.860,	0.888, 0.880, 0.880, 0.920, 0.900, 0.920	
								DT							0.910, 0.900, 0.870, 0.910, 0.910, 0.900	0.790, 0.820, 0.820, 0.840, 0.840, 0.840	
								LR							0.910, 0.920, 0.920, 0.920, 0.920, 0.910	0.820, 0.860, 0.860, 0.860, 0.900, 0.900	
								KNN							0.910, 0.880, 0.880, 0.900, 0.880, 0.850	0.860, 0.900, 0.900, 0.920, 0.920, 0.960	
								SVM							0.910, 0.900, 0.880, 0.930, 0.910, 0.920	0.840, 0.900, 0.900, 0.880, 0.900, 0.900	
			42.0 ± 13.0	MRI	24	T1W (MS)	NS	LDA	Diagnosis of CM1 in Independent Cohort	2	CM1/healthy	4-Fold-Cross-Validation	24	0.960, 0.970, 0.960, 0.940, 0.960, 0.960	N/A	N/A	N/A
								QDA						0.930, 1.00, 0.970, 0.960, 0.920, 0.960			
								NB						0.960, 0.960, 0.930, 0.930, 0.960, 0.960			
								DT						0.930, 0.920, 0.880, 0.920, 0.920, 0.920			
								LR						0.960, 0.960, 0.960, 0.960, 0.960, 0.960			
								KNN						0.980, 0.830, 0.850, 0.910, 0.940, 0.960			
								SVM						0.960, 0.960, 0.880, 0.960, 0.960, 0.960			
Tanaka et al. [28]	2022	212	30 yrs	MRI	212	T1W-FLAIR (S)	R and NS	ResNet50 (Deep CNN) dropout rate 0.5	DIAGNOSIS OF CM1	2	sCM1/control	10-Fold-Cross-Validation	43	N/A	0.864	0.714	0.890
															0.818	0.905	0.930
															0.864	0.952	0.980
															0.818	0.952	0.960
								VGG19 (Deep CNN) dropout rate 0.5						N/A	0.864	0.905	0.970
															0.773	1.00	0.980
															0.864	1.00	1.00
															0.773	1.00	0.940
								VGG19 (Deep CNN) dropout rate 0.8						N/A	0.818	1.00	1.00
															0.773	1.00	0.970
															0.955	1.00	1.00
															0.727	1.00	0.950
								VGG 19 (Deep CNN) + Augmentation						N/A	0.971	0.974	0.992
								ResNet50 (Deep CNN) + Augmentation							0.940	0.944	0.983

No. = Number; N/A = Not Applicable; MS = Midsagittal; D.R = Diagnostic Reference; NS = Neurosurgeon(s); R = Radiologist; E = Expert; CNN = Convoluted Neural Network; XGB = Extreme Gradient Boosting (XGBoost); SGB = Stochastic Gradient Boosting; BC = Bagged CART; RF = Random Forest; LR = Logistic Regression; LDA = Linear Discriminant Analysis; QDA = Quadratic Dominant Analysis; NB = Naïve Bayes; BT = Binary Trees; DT = Decision Trees; KNN = K-Nearest Neighbors; SVM = Support Vector Machine; ResNet50 = A CNN Machine Learning Algorithm; VGG19 = A CNN Machine Learning Algorithm; C = Combinations (Machine Learning Models that utilized combinations of morphometric parameters); S = Single(Machine Learning Models that utilize only a single morphometric parameter as input data); CM1 = Chiari Malformation Type-1; sCM1 = Significant Chiari Malformation Type-I (CM1 requiring surgery, due to symptoms or syringomyelia); Acc = Accuracy; Sen = Sensitivity; Spe = Specificity; AUC = Area Under Curve.

**Table 2:** The table demonstrates the included studies focusing on the prognosis of patients with Chiari Malformation.

Author	Year	N	Age	Imaging Modality	No. of Images	SEQ	D.R	ML Model	Output Classes	Outcome	Validation	Testing Set	Acc	Sen	Spe	AUC/ AUROC
El-Hajj et al. [29]	2024	7106	9.2 yrs	N/A	N/A	N/A	N/A	LR	2	Readmission/No-Readmission After PFD	10-Fold-Cross-Validation	1403	N/A	N/A	N/A	0.761
								GNB								0.741
								KNC								0.921
								DTC								0.837
								RFC								0.960
								LR	2	Reoperation/No-Reoperation After PFD						0.820
								GNB								0.745
								KNC								0.944
								DT								0.916
								RF								0.990
King et al. [30]	2024	57	31 yrs	MRI	N/A	T1-FLAIR (S) and CPC MRI	NR and NS	CLAM (DL) + ResNet50 (CNN)	2	Improvement/Recurrence	5-Fold-Cross-Validation	11	0.647	0.725	0.483	0.680
								LR	2	Improvement/Recurrence			0.635	0.65	0.600	0.610
													0.611	0.6	0.633	0.690
													0.406	0.475	0.233	0.380
													0.529	0.525	0.533	0.660
													0.402	0.45	0.283	0.340
													0.473	0.550	0.300	0.400
													0.668	0.675	0.650	0.710
													0.665	0.675	0.633	0.710
Thakar et al. [31]	2020	82	adults (N/A)	MRI	N/A	N/A	N/A	SVM	2	Improvement/No Improvement (CCOS Score)	K-Fold-Cross-Validation	57	0.820	N/A	N/A	0.870
								RF					0.700			0.820
								XGB					0.840			0.880
Mesin et al. [32]	2022	58 pts	9.71 yrs	MRI	58	T1W (S)	NS	SVM (M) x3	2	Improvement/Not Improvement	Leave-One-Out	5	0.820	N/A	N/A	N/A
								SVM (A) x3					0.710			

No. = Number of Patients; N/A = Not Applicable; (S) = Sagittal; CPC = Cine Phase-Contrast; D.R = Diagnostic Reference; NS = Neurosurgeon; NR = Neuroradiologist; LR = Logistic Regression; GNB = Gaussian Naïve Bayes; KNC = K-Neighbors Classifier; DT = Decision Tree; RF = Random Forest; DL = Deep Learning; SVM = Support Vector Machine; XGB = Extreme Gradient Boosting (XGBoost); CLAM = Clustering-Constrained Attention Multiple Instance Learning; ResNet50 = A CNN Machine Learning Algorithm; M = Manually; A = Automatically; x3 = Mesin et al. developed 3 SVM models in each category; PFD = Posterior Fossa Decompression surgery; CCOS = The Chicago Chiari Outcome Score; Acc = Accuracy; Sen = Sensitivity, Spe = Specificity; AUC/AUROC = Area Under Cur.

**Table 3:** The table demonstrates a comprehensive classification of the diagnostic ML models per category and performance.

Category of Machine Learning	Model	Sensitivity	Specificity	Ref.
Deep Learning-based models	CNN	0.920–0.970	1.00	[25]
	ResNet50	0.818–0.864	0.714–0.952	[28]
	ResNet50 + Augmentation	0.940	0.944	
	VGG19	0.727–0.955	0.905–1.00	
	VGG19 + Augmentation	0.971	0.974	
Tree-based models	XGBoost	N/A	N/A	[26]
	SGB	N/A	N/A	
	BC	N/A	N/A	
	RF	0.690–1.00	0.920–1.00	
	DT	0.870–0.910	0.790–0.840	[27]
Statistical-based models	LR	0.910–0.920	0.790–0.840	[26]
	LDA	0.910–0.930	0.820–0.880	
	QDA	0.910–0.950	0.820–0.880	
	KNN	0.850–0.910	0.860–0.960	
	NB	N/A	N/A	
	KNN	N/A	N/A	
Kernel-based models	SVM	0.880–0.930	0.840–0.900	[27]
Hybrid models	NB-BT	0.880	0.848	[24]

CNN = Convoluted Neural Network; XGBoost = Extreme Gradient 150 Boosting; SGB = Stochastic Gradient Boosting; BC = Bagged CART; RF = Random Forest; LR = Logistic Regression; LDA = Linear Discriminant Analysis; QDA = Quadratic Dominant Analysis; NB = Naïve Bayes; BT = Binary Trees; DT = Decision Trees; KNN = K-Nearest Neighbors; SVM = Support Vector Machine; ResNet50 = A CNN Machine Learning Algorithm; VGG19 = A CNN Machine Learning Algorithm; Ref = Reference; N/A = Not available.

**Table 4:** The table demonstrates a comprehensive classification of the prognostic ML models per category and performance.

Category of Machine Learning	Model	AUC/AUROC	Ref.
Tree-based models	DT	0.837–0.916	[29]
	RF	0.820–0.990	[29,31]
	XGBoost	0.880	[31]
Statistical-based models	LR	0.340–0.820	[29,30]
	KNC	0.921–0.940	[29]
	GNB	0.741–0.745	
Kernel-based models	SVM	0.870	[31,32]
Hybrid models	CLAM + ResNet50	0.680	[30]

LR = Logistic Regression; GNB = Gaussian Naïve Bayes; KNC = K-Neighbors Classifier; DT = Decision Tree; RF = Random Forest; SVM = Support Vector Machine; XGB = Extreme Gradient Boosting (XGBoost); CLAM = Clustering-Constrained Attention Multiple Instance Learning; ResNet50 = A CNN Machine Learning Algorithm; Ref = References.

## Supplementary Materials

The following supporting information can be downloaded at: https://www.mdpi.com/article/10.3390/children12020244/s1, Table S1. This table demonstrates the performance metrics of ML models as calculated from independent cohorts by Urbizu et al. [27]. Table S2. This table demonstrates the input data utilized by each model in the studies included in our review and the surgical procedures performed.
